# Hyper‐acetylation contributes to the sensitivity of chemo‐resistant prostate cancer cells to histone deacetylase inhibitor Trichostatin A

**DOI:** 10.1111/jcmm.13475

**Published:** 2018-01-12

**Authors:** Qingqing Xu, Xiaofei Liu, Shiqin Zhu, Xuelei Hu, Huanmin Niu, Xiulei Zhang, Deyu Zhu, Effat Un Nesa, Keli Tian, Huiqing Yuan

**Affiliations:** ^1^ Department of Biochemistry and Molecular Biology Shandong University School of Medicine Jinan China

**Keywords:** chemo‐resistant mCRPC, HDAC inhibitors, apoptosis, hyper‐acetylation, acetyl‐CoA, ER stress

## Abstract

Therapeutic agents are urgently needed for treating metastatic castration‐refractory prostate cancer (mCRPC) that is unresponsive to androgen deprivation and chemotherapy. Our screening assays demonstrated that chemotherapy‐resistant prostate cancer (PCa) cells are more sensitive to HDAC inhibitors than paired sensitive PCa cells, as demonstrated by cell proliferation and apoptosis *in vitro* and *in vivo*. Kinetic study revealed that TSA‐induced apoptosis was significantly dependent on enhanced transcription and protein synthesis in an early stage, which subsequently caused ER stress and apoptosis. ChIP analysis indicated that TSA increased H4K16 acetylation, promoting ER stress gene transcription. The changes in Ac‐H4K16, ATF3 and ATF4 were also validated in TSA‐treated animals. Further study revealed the higher enzyme activity of HDACs and an increase in acetylated proteins in resistant cells. The higher nucleocytoplasmic acetyl‐CoA in resistant cells was responsible for elevated acetylation status of protein and a more vigorous growth state. These results strongly support the pre‐clinical application of HDAC inhibitors for treating chemotherapy‐resistant mCRPC.

## Introduction

Docetaxel is the only chemotherapeutic with the proven survival benefit for treating metastatic castration‐resistant prostate cancer (mCRPC) 1. However, docetaxel unexceptionally results in frequent side effects and causes multidrug resistance (MDR) 2. Several mechanisms have been proposed in docetaxel resistance including mutations in drug binding sites on tubulin, the inappropriate gene expression resulting from epigenetic changes in histones and chromatin structures, overexpression of the ATP‐dependent efflux proteins and elevation of pro‐inflammation factors 1, 2, 3. Elucidation of the mechanisms for gene expressions has shed light on the importance of acetylation–deacetylation of histones mediated by histone acetyltransferases (HATs) and histone deacetylases (HDACs). Modulation of the HDAC enzymes alters the expression of proto‐oncogenes and tumour suppressor genes, regulating the potential neoplastic activity of the cells. Studies have shown that HDACs are abundantly expressed and involved in regulation of several important proteins, for example, the androgen receptor (AR) that is critical in CRPC development 4, 5. Therefore, histone deacetylase inhibitors (HDACi) emerge as important therapeutic agents in CRPC 6. For example, trichostatin A (TSA) shows the general inhibition of class I, II and IV HDACs with nanomolar potency 6, resulting in hyper‐acetylation of chaperones, impairing chaperone activity and causing the endoplasmic reticulum (ER) stress and apoptosis 7. TSA and suberoylanilide hydroxamic acid (SAHA), another HDACi, also were capable of inducing more dramatic apoptosis and reducing efflux transport protein P‐gp expression in the drug‐resistant cells compared to their parental counterparts. This aspect was marked in the P‐gp‐expressing daunomycin‐resistant L1210R, P388R (murine leukaemia) and adriamycin‐resistant MCF‐7/ADR (human breast carcinoma) cell lines and less noticeable in the MRP‐expressing HL60/R cell line 8.

In addition, the combination of HDACi and other therapeutic approaches shows a synergistic effect on reversing resistance development and improving the sensitivity of PCa cells to chemotherapy 9, 10.

In this study, we report a novel finding that chemotherapy‐resistant PCa cells with higher levels of acyl‐CoA and acetylated proteins were more sensitive to TSA/SAHA than the paired parent cells. TSA facilitated cell death *via* exacerbating acetylation and enhancing in the gene expression, which led to inducing ER stress in resistant cells with active metabolic processes.

## Materials and methods

### Cell culture and treatments

Prostate cancer PC3 cells obtained from the Cell Bank of the Chinese Academy of Sciences (Shanghai, China) and docetaxel‐resistant PC3/Doc cells, as previously described 11, lung adenocarcinoma H460 and paclitaxel‐resistant H460/RT cells, oral epithelium carcinoma KB cells and the vincristine‐resistant KB/VCR cells, murine PCa RM‐1 cells (The Cell Bank of Chinese Academy of Sciences) and RM‐1/Doc cells (docetaxel‐resistant cell line derived from RM‐1) were cultured in RPMI 1640 medium (HyClone, Logan, UT, USA) supplemented with 10% foetal bovine serum (GIBCO, Grand Island, NY, USA), 100 U/ml penicillin and 100 g/ml streptomycin.

Trichostatin A (TSA), suberoylanilide hydroxamic acid (SAHA), the PI3K inhibitor LY294002, cycloheximide (CHX), actinomycin (Act D) and sodium tauroursodeoxycholate (TUDCA) were purchased from Sigma‐Aldrich (St‐Louis, MO, USA). The pan‐caspase inhibitor Z‐VAD‐fmk was obtained from Enzo Life Sciences (Plymouth Meeting, PA, USA).

In some experiments, the cells were exposed to z‐VAD‐fmk, CHX, LY294002 or Act D for 2 hrs before TSA treatment. DMSO was used as the control vehicle.

### Cell viability and cell death assay

Cell viability was determined *via* a 3‐(4, 5‐dimethylthiazol‐2‐yl)‐2, 5‐diphenyl‐2H‐tetrazolium bromide (MTT, Sigma‐Aldrich) assay on a plate reader (Bio‐Rad, Hercules, CA, USA). Cell death was measured by propidium iodide (PI) and annexin V‐FITC staining with flow cytometry (BD Biosciences, San Jose, CA, USA).

### 5‐ethynyl‐2′‐deoxyuridine (EdU) incorporation assay

PC3 and PC3/Doc cells were treated with TSA and 10 μM EdU; 16 hrs after treatment, EdU incorporation assay was carried out using the Cell‐Light EdU imaging detecting kit (Millipore, German) according to the manufacturer's instructions. EdU is an alternative thymidine analogue whose incorporation can be used to label and identify cells undergoing DNA replication. EdU‐positive cells were calculated with (EdU add‐in cells/DAPI‐stained cells) ×100%.

### Western blot assay

After transfection and/or treatment with chemicals, the cells were lysed for a Western blot assay as described previously 12. The blots were incubated with primary antibodies against PERK, p‐PERK (Thr981), ATF4 (CREB‐2), ATF3, Bcl‐2, BAX, poly (ADP‐ribose) polymerase (PARP), HDAC1 glyceraldehyde 3‐phosphate dehydrogenase (GAPDH) (Santa Cruz Biotechnology, Dallas, TX, USA), cleaved caspase‐3 (Epitomics, Burlingame, CA, USA), mTOR and phospho‐mTOR (Ser2448), DJ‐1, GRP78, eIF2a, phospho‐eIF2a, AKT, phospho‐AKT (Ser473), HDAC5, HDAC6 (Cell Signaling Technology, Danvers, MA, USA), 4EBP1 (Abcam, Cambridge, MA, USA), HDAC2, HDAC4 and COX4 (Proteintech, Wuhan, China) overnight at 4°C, respectively, followed by appropriate peroxidase‐conjugated secondary antibodies. GAPDH or actin served as an internal control. The detection system visualization (Millipore) was followed by exposure to X‐ray film.

### RT‐PCR and qRT‐ PCR analysis

Total RNA was obtained using TRIzol reagent (TaKaRa) and reverse transcribed to cDNA using a RrimeScript^TM^ RT reagent kit (TaKaRa, China). qPCR was performed using the Eppendorf qRT‐PCR System. Changes in the mRNA levels of desired genes were normalized to the level of 18s. Data were analysed using the 2^−▵▵*ct*^ method. Amplified products according to RT‐PCR protocol were run agarose gel electrophoresis, with ultraviolet scanning. GAPDH served as an internal control. The primer sequences are shown in Table S1.

### Transient transfection of plasmids and siRNAs

PC3/Doc cells were transiently transfected with dominant‐negative PCMV5‐AKT1‐K179M (AKT1‐DN), ATF3 siRNA (described previously 13) or HDAC5 siRNA (sc‐35542) (Santa Cruz Biotechnology) using lipofectamine 2000 (Invitrogen, Carlsbad, CA, USA). Empty vectors PCMV5 served as controls. After 24‐hrs transfection, cells were treated with TSA or vehicle for an additional 24 hrs, and cell lysates were subjected to a Western blot assay. Cell viability and death were determined by MTT assays.

### ChIP

Chromatin immunoprecipitation (ChIP) analysis was performed according to the Keji Zhao published protocol using MNase 14. The product was incubated with anti‐histone H4K16 acetylation (GC‐132) (PTM Biolabs, Hangzhou, China) or anti‐human IgG. The antibody‐bound complex was precipitated by protein A‐Sepharose beads (Santa Cruz Biotechnology) for regular PCR. The primer sequences are shown in Table S2.

### Murine homograft study

RM‐1 cells from C57BL/6 murine prostate tumours, which are androgen independent and commonly used to develop homograft animal models 15, 16, were included to establish multidrug‐resistant RM/Doc cell lines by docetaxel exposure. The inhibitory effect of TSA on tumour growth was evaluated in the RM‐1 and RM/Doc homograft models. Male C57BL/6 mice (4–5 weeks old) were obtained from the Experimental Animal Center of Shandong (Shandong, China). The cells (1 × 10^5^) in 0.1 ml of physiological saline were subcutaneously injected into the right flanks of the mice and allowed to establish tumours. After 1 week, the animals were pair‐matched by tumour size and treated by s.c. injection of 1 mg/kg TSA (dissolved in 1% DMSO, 5% TWEEN‐80) or 5 mg/kg docetaxel. The injections were performed every 2 days for 2 weeks. The tumour volume and animal weight measurements were recorded. The volume (in mm^3^) was calculated from the formula 0.5 × L × W^2^ (L = length, W = width). At the end of the experiment, all tumours were resected for Western blotting and immunohistochemistry. All animal experiments were approved by the Ethics Committee of Shandong University School of Medicine (Permit Number: LL‐201602073) and conducted accordingly.

### Statistical analysis

Data are presented as the mean ± S.D. For comparison of the significant differences more than two groups, one‐way anova tests were used, while other comparisons were performed with Student's unpaired *t*‐test using GraphPad Prism software (San Diego, CA, USA). *P *<* *0.05 was considered to be significant (*), and *P *<* *0.001 was highly significant (***).

## Results

### Drug‐resistant PC3/Doc cells are sensitive to HDAC inhibitors

A screening assay was initially performed to identify the chemical that potently inhibited cell proliferation in drug‐resistant PC3/Doc cells. The concentration of chemicals that induced cell death in 50% (IC50) is summarized in Table 1. The results showed that the IC50 of TSA was much lower in resistant PC3/Doc cells (0.33 ± 0.06 μM *versus* 2.87 ± 0.21 μM in PC3 cells; Table 1 and Fig. S1E). Consistent with the observations above, PC3/Doc cells were also sensitive to another HDAC inhibitor SAHA, but to a less degree (2.81 ± 0.36 μM *versus* 5.99 ± 0.72 μM; Table 1 and Fig. S1F). These results demonstrated that HDACi, particularly TSA, functioned as a potent cytotoxic agent in resistant PC3/Doc cells.

**Table 1:** Effect of chemotherapy drugs on docetaxel‐sensitive and docetaxel‐resistant cell lines

IC50 (μM)	PC3	PC3/Doc
Doxorubicin	4.24 ± 0.57	9.15 ± 0.36
Vincristine	10.03 ± 0.21	14.80 ± 0.12
Cisplatin	6.16 ± 0.51	6.63 ± 0.53
Docetaxel (nm)	6.08 ± 0.73	23.37 ± 0.18
Trichostatin A	2.87 ± 0.21	0.33 ± 0.06
SAHA	5.99 ± 0.72	2.81 ± 0.36

**Figure 1:**
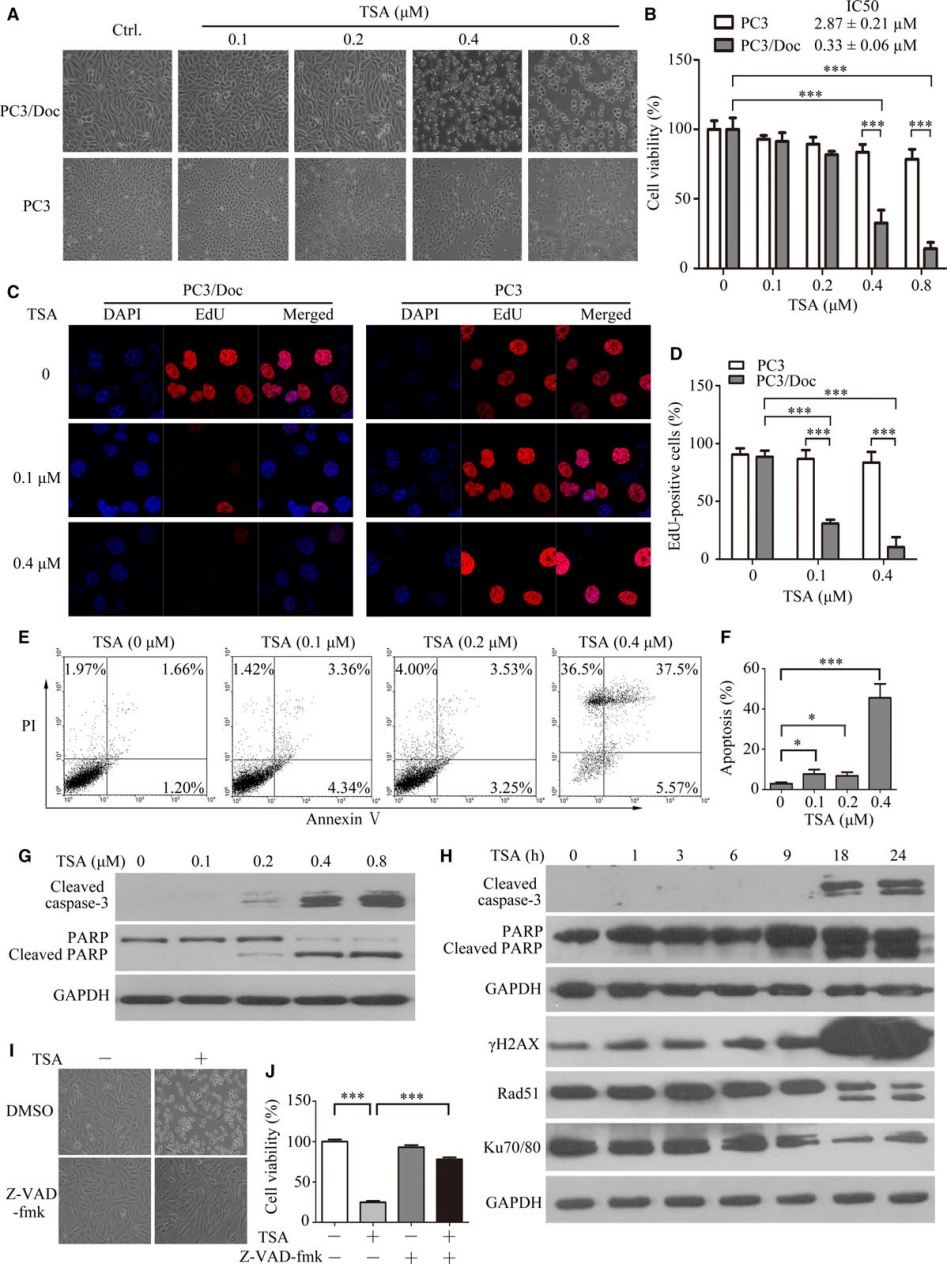
Effect of TSA on docetaxel‐sensitive and docetaxel‐resistant PCa cells. (**A**) The morphological changes after 24‐hrs treatment with 0, 0.1, 0.2, 0.4 and 0.8 μM of TSA in PC3 and PC3/Doc cells. (**B**) Cell viability was determined by the MTT assay after treatment with different TSA concentrations for 24 hrs in PC3 and PC3/Doc cells. (**C**) Cell proliferation was examined by EdU incorporation assay at 16 hrs after TSA treatment, and (**D**) EdU‐positive cells were calculated. (**E**) Detection of apoptotic cells after annexin V/PI staining by flow cytometric analysis after treatment with 0, 0.1, 0.2 and 0.4 μM of TSA for 24 hrs in PC3/Doc cells and (**F**) the population of the apoptotic cell was calculated. (**G**) Cleaved caspase‐3 and PARP expression were examined for cells treated with TSA at various concentrations for 24 hrs. (**H**) Western blot analysis of the cleaved caspase‐3, PARP, rH2AX, Rad 51 and Ku70/80 in PC3/Doc cells treated with TSA (0.4 μM) at different time‐points. (**I**) Morphological changes and (**J**) cell viability in the absence or presence of pan‐caspase inhibitor (z‐VAD‐fmk). PC3/Doc cells were exposed to 10 μM z‐VAD‐fmk for 2 hrs prior to TSA (0.4 μM) or vehicle treatment for 24 hrs. *P <* *0.05, ***P <* *0.001.

Morphological changes displayed that TSA was able to cause apoptosis in PC3/Doc cells at 0.4 μM (Fig. 1A). A rapid decrease (67.4 ± 10.73%) in cell viability was noted when cells exposed to 0.4 μM TSA, which was up to 85.82 ± 8.94% inhibited with 0.8 μM TSA (Fig. 1B). EdU incorporation assays showed that 0.4 μM TSA significantly suppressed cell proliferation in drug‐resistant PC3/Doc cells, but had less inhibitory effect in PC3 cells at the same dose (Fig. 1C, D), supporting that resistant PC3/Doc cells were more sensitive to TSA. Flow cytometry analysis revealed that the percentages of apoptotic cells were approximately 7.70%, 6.78% and 43.07% after treatment with 0.1, 0.2 and 0.4 μM of TSA, respectively. We noted that TSA also caused necrosis in cells treated at higher concentrations (Fig. 1E, F).

Further investigation showed that TSA treatment did not affect the expression of pro‐apoptotic BAX and anti‐apoptotic Bcl2 in PC3/Doc cells (Fig. S1G). However, cleaved caspase‐3 and PARP cleavage, as an indicator of caspase activation, initiated in PC3/Doc cells exposed to 0.2 μM TSA, which sustained with increasing concentrations (Fig. 1G). Time kinetic analysis (Fig. 1H) revealed that in PC3/Doc cells caspase‐3 activation and PARP cleavage were observed at 18 hrs and increased with longer treatment with 0.4 μM TSA. However, TSA was unable to cause detectable PARP cleavage and cell death in PC3 cells even at 1 μM (Fig. S1H). In addition, we examined the effect of SAHA on PARP cleavage to elucidate if this change is common in HDACi. Treatment of PC3/Doc cells with SAHA also significantly induced a PARP cleavage after prolonged treatments (Fig. S1I), consistent with the observations of TSA on PARP cleavage (Fig. 1G). Moreover, changes in the phosphorylation of H2AX (γ‐H2AX), a DNA damage marker, kept pace with apoptosis induction, whereas Rad51 and Ku70/80, which are induced upon DNA damage, proportionally decreased with longer TSA treatment (Fig. 1H). The results in Figure 1I, J provided evidence that cell death induced by TSA was nearly restored in the presence of z‐VAD‐fmk, a pan‐inhibitor of caspases. Together, the data indicated that chemo‐resistant PC3/Doc cells were sensitive to HDAC inhibitors, and 0.33 μM (IC50) TSA efficiently triggered cell death *via* caspase‐dependent apoptosis.

### TSA exhibits potent antitumour activity in drug‐resistant homograft mice

**Figure 2:**
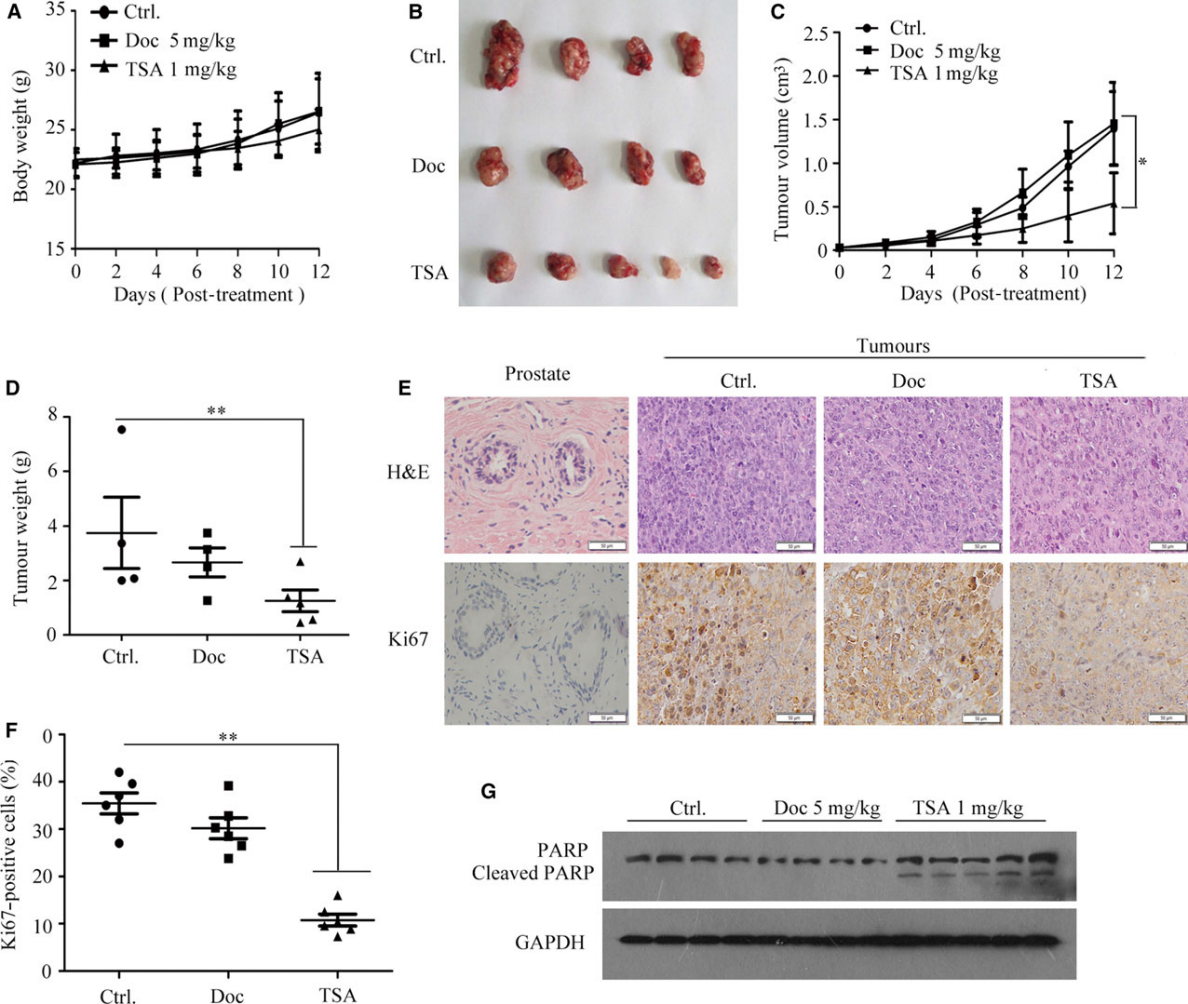
TSA exhibits potent antitumour activity in the drug‐resistant homograft mice. (**A**) The mice body weight of the three groups was measured every 2 days after the indicated treatment. (**B**) Representative tumours from the three groups are shown (TSA group: *n *=* *5; Ctrl and Doc group: *n *=* *4). (**C**) Tumour volume from homografts in different treatment groups was recorded every 2 days. Data are represented as the mean ± S.E.M. (TSA group: *n *=* *5; Ctrl and Doc group: *n *=* *4). *P *<* *0.05 compared with the negative control. (**D**) Tumour weight was detected at the time of killing for different treated groups. Data are shown as the mean ± S.E.M. (TSA group: *n *=* *5; Ctrl and Doc group: *n *=* *4). (**E**) Representative images of H&E staining and immunohistochemical staining of the different treated groups. (**F**) Ki67‐positive rates in each group. **P <* *0.01 compared with the negative control. Data are shown as the mean ± S.E.M. (**G**) Western blot analysis of PARP expression in differentially treated groups.

Driven by these observations, we evaluated the antitumour efficiency of TSA *in vivo* prior to mechanical investigation. Animals were randomly assigned to each group (*n *=* *5). Three animals failed to complete the study: one in the docetaxel group, one control in the drug‐resistant RM/Doc group and one in the RM‐1 control. As shown in Figure 2A, after 12 days of treatments, the initial and final body weights in tumour‐bearing mice remained almost unchanged by the administration of docetaxel or TSA compared with the control group. The tumours arising from the control mice grew to an average size of 1395.45 ± 523.72 mm^3^ (Fig. 2B, C). Docetaxel treatment (5 mg/kg) had no significant effect on RM/Doc tumour growth in terms of the volume (1254.09 ± 472.86 mm^3^). However, the tumour size (538.57 ± 349.56 mm^3^) markedly decreased in the TSA group, and growth inhibition was 61.3% (Fig. 2B, C). Similarly, the tumour weights were highest in the control (3.75 ± 2.60 g), decreased to 2.67 ± 1.06 g in the docetaxel‐treated mice and 1.25 ± 0.90 g in the TSA group (Fig. 2D). These results provided fundamental evidence that docetaxel had little inhibitory effect on chemo‐resistant tumour growth, whereas the effect of TSA was clearly demonstrated. In addition, docetaxel antitumour activity was validated in mice bearing RM‐1 cells with 64.03% inhibition (Fig. S1I–L), supporting the observations that docetaxel was effective for PCa, but to the less extent for drug‐resistant PCa. Regarding the antitumour efficacy of TSA on resistant PCa, Figure 2E, F indicated that TSA noticeably inhibited cell proliferation, as indicated by positively stained Ki67 cells (10.79 ± 3.22% *versus* 35.43 ± 5.40% in control group), while the Ki67 positivity was 30.2 ± 5.39% in docetaxel‐treated group, supporting the observations that TSA potently inhibited cell proliferation (Fig. 1C). Consistent with the results in cultured cells (Fig. 1), PARP cleavage was predominantly increased in mice that were given TSA, and no cleaved PARP was observed in tumours treated with docetaxel (Fig. 2G). Thus, TSA acted as a potent antitumour agent, inhibiting chemo‐resistant PCa cell proliferation and inducing apoptosis *in vitro* and *in vivo*.

### TSA induces apoptosis associated with ER stress activation by affecting gene expression

**Figure 3:**
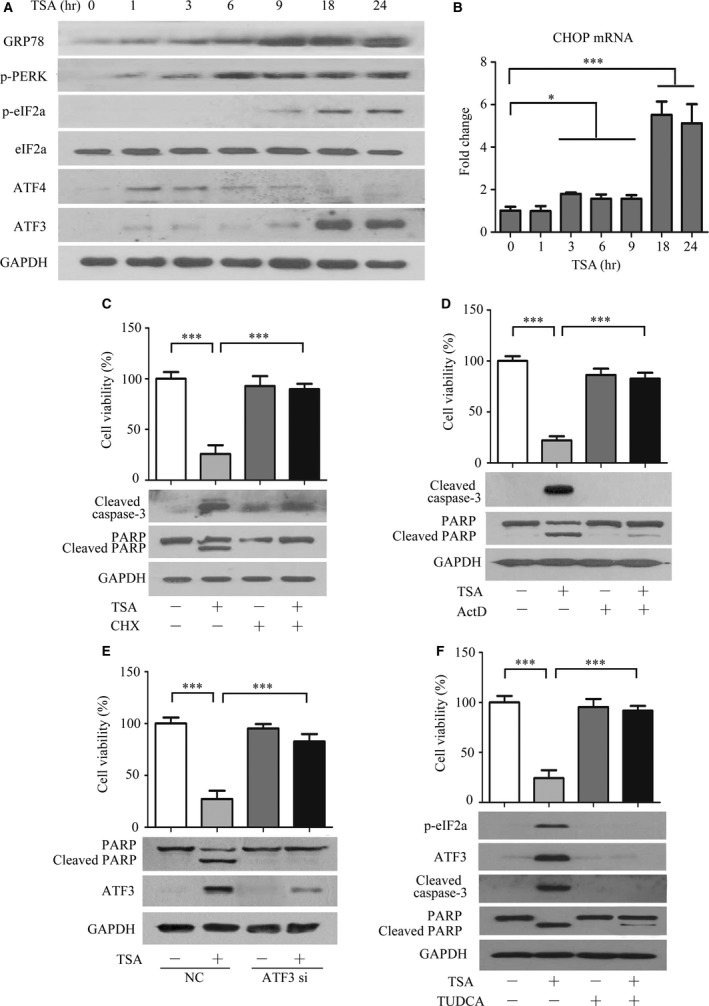
TSA causes ER stress by influencing protein synthesis. (**A**) Analysis of the effect of TSA on proteins associated with ER stress by Western blot. (**B**) Measurement of CHOP mRNA expression in PC3 cells exposed to TSA by real‐time RT‐PCR. *P <* *0.05, ***P <* *0.001, compared with the control (0‐hr treatment). Data shown are the mean ± S.D., *n *=* *3. (**C**,** D**) The protein synthesis inhibitor CHX and transcription inhibitors Act D attenuated TSA‐mediated cell death. PC3/Doc cells were incubated (**C**) with CHX (2 μg/ml) or (**D**) with Act D (4 μM) for 2 hrs prior to TSA treatment of 24 hrs. Cell viability was measured by the MTT assay, and cleaved caspase‐3 and PARP expression were determined by Western blot analysis. Values are the mean ± S.D. (*n *=* *3). ***P <* *0.001, *versus* the vehicle control group. (**E**) Impact of knock‐down of ATF3 by siRNA on the cell viability of TSA‐treated PC3/Doc cells, and cleaved PARP and ATF3 expression were determined by Western blot analysis. PC3/Doc cells were transfected with siRNAs for 24 hrs prior to TSA treatment. (**F**) The ER stress inhibitor TUDCA reverted TSA‐mediated cell death. PC3/Doc cells were incubated with TUDCA (100 μM) for 2 hrs prior to TSA treatment of 24 hrs. Cell viability was measured by the MTT assay, and cleaved caspase‐3 and PARP expression were determined by Western blot analysis. Values are the mean ± S.D. (*n *=* *3). ***P <* *0.001 *versus* the vehicle control group.

We next investigated the action mechanism by which TSA facilitated cellular apoptosis. Firstly, the activity of caspase‐8 that is involved in the death receptor pathway remained in an extremely low state and slightly increased at 24 hrs (Fig. S2A), suggesting that death receptor pathway might not be critical in TSA‐mediated cells death. The results in Figure S2B–D showed that the levels of reactive oxygen species (ROS) and DJ‐1 (an oxidative stress‐response protein), thioredoxin‐binding protein‐2 (TBP‐2, a principal antioxidant scavenger of ROS) 17, remained unchanged in response to TSA in resistant PC3/Doc cells. Considering the role of HDAC in the regulation of gene expression, together with observations that TSA‐induced apoptosis occurred following longer exposure (Fig. 1E), we, therefore, tested the possibility that gene expression was initially enhanced by TSA, subsequently leading to the induction of ER stress and promoting cell death 18. Figure 3A showed that GRP78, a sensor of ER stress, appeared at 1 hr and was evident after 9‐hrs treatments. Increased phospho‐PERK was noted at 3 hrs and steadily increased with time. Similar results were observed in cells exposed to SAHA (Fig. S2G). Accordingly, phosphorylation of eIF2a, which is a substrate of PERK 19, 20 and impairs global translation, was up‐regulated at 9 hrs and maintained at high levels following longer treatments; the total eIF2a remained unchanged. We also looked at transcription factor 4 (ATF4), which is critical in the transcriptional regulation of ER stress‐responsive gene expression. ATF4 was up‐regulated after 1 hr and gradually decreased by 24 hrs (Fig. 3A), supporting that TSA‐initiated gene expression was an early event. In addition, upon exposure to TSA, ATF3 and CHOP/GADD153 expressions were marginally increased up to 9 hrs and sharply elevated after 18‐hrs treatments (Fig. 3A, B). These findings indicated that apoptotic markers were enhanced following elicitation of ER stress with TSA exposure, which was in agreement with the results in Figure 1E.

As the elevated phospho‐eIF2a presented after longer TSA treatments, protein synthesis likely contributed to TSA‐induced ER stress and apoptosis. To address this question, cells were pre‐treated with protein synthesis inhibitor CHX or transcription inhibitor Act D prior to TSA treatment. The results indicated that TSA‐mediated cell death was predominantly reversed in the presence of CHX, accompanied by a reduction in caspase‐3 activation and PARP cleavage (Fig. 3C). Similar results were observed after pre‐treatment with Act D (Fig. 3D). To validate this finding, we genetically silenced ATF3 by siRNA due to its important roles in gene transcription upon ER stress. ATF3 depletion dramatically reverted TSA‐mediated cell death and viability suppression, associated with the alleviation of the PARP cleavage (Fig. 3E). Moreover, PC3/Doc cells pre‐treated with 100 μM TUDCA, an ER stress inhibitor; the cell death induced by TSA was significantly restored; and phosphor‐eIF2α and ATF3 induction were attenuated, leading to the reduction in caspase‐3 activation and PARP cleavage (Fig. 3F). Thus, TSA stimulated apoptosis through eliciting ER stress, which might be ascribed to a TSA‐induced enhancement of gene expression.

### TSA affects protein synthesis through modulating AKT/mTOR/4EBP1 signalling

**Figure 4:**
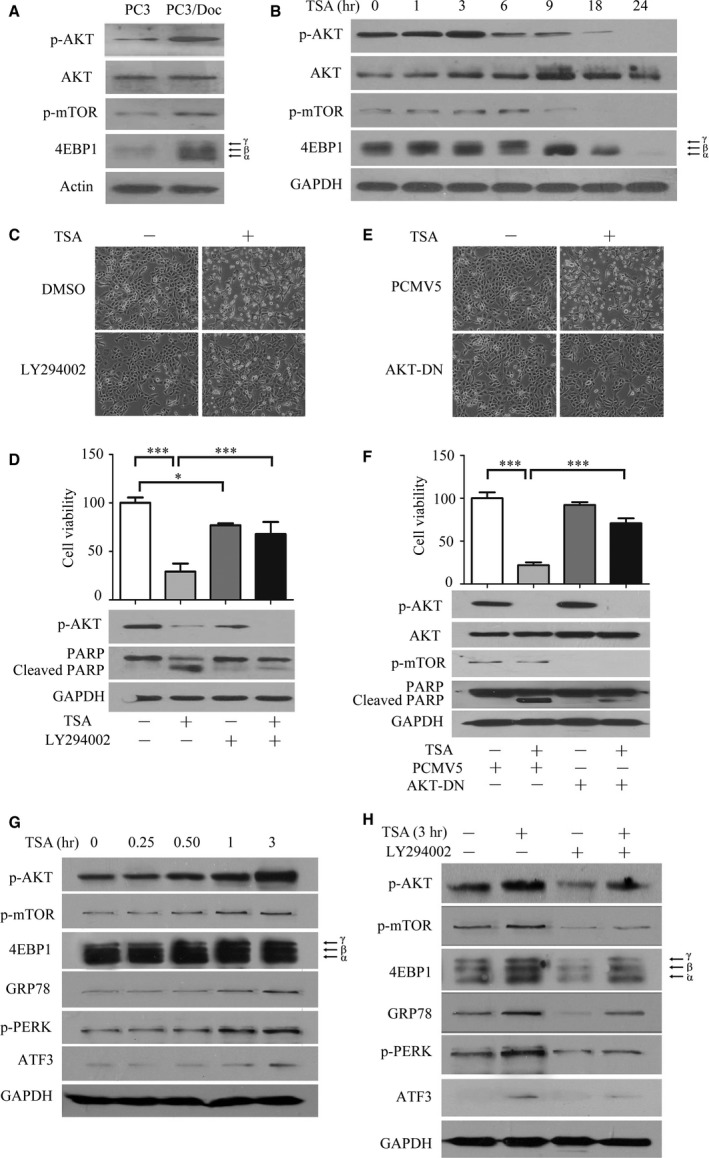
TSA affects protein synthesis through modulating AKT/mTOR/4EBP1 signalling. (**A**) Western blot analyses of the expression differences of the AKT/mTOR/4EBP1 pathway between PC3 and PC3/Doc cells. (**B**) Analysis of the effect of TSA on proteins associated with protein synthesis by Western blot analysis. (**C**,** D**) The PI3K inhibitor LY294002 attenuated TSA‐mediated cell death. PC3/Doc cells were incubated with LY294002 (20 μM) for 2 hrs prior to 0.4 μM TSA treatment. Morphological changes were observed under a light microscope, cell viability was measured by the MTT assay, and cleaved caspase‐3 and PARP expression were determined by Western blot analysis. (**E, F**) PC3/Doc cells transfected with AKT1‐DN for 24 hrs prior to 0.4 μM TSA treatment. Cell viability was investigated by MTT (upper panel), and cell death was determined by Western blot analysis. *P <* *0.05, ***P <* *0.001. Data shown are the mean ± S.D., *n *=* *3. (**G**) Western blot analyses of the effect of AKT/mTOR signalling and proteins associated with ER stress when exposed to TSA in 3 hrs. (**H**) PC3/Doc cells were incubated with LY294002 for 2 hrs prior to 3‐hrs TSA treatment. The expression of AKT/mTOR signalling and proteins associated with ER stress were determined by Western blot analysis.

Given the importance of protein synthesis in TSA‐induced cell death, we next investigated the role of the AKT/mTOR/4EBP1 signalling pathway, which is the dominant mechanism controlling protein synthesis. As shown in Figure 4A, compared to PC3 cells, the phospho‐AKT and phospho‐mTOR levels were significantly up‐regulated in PC3/Doc cells. Phospho‐4EBP1 (γ‐band, inactivation of 4EBP1 by mTORC1‐mediated phosphorylation) that enhances translation initiation complex formation and cap‐dependent translation 21 was more evident after treatment (Fig. 4A). These observations supported that the enhancement of cellular metabolisms, including protein synthesis, is associated with drug resistance 2.

Kinetic study revealed that, upon TSA treatment, phospho‐AKT started to increase after 1 hr, peaked at 3 hrs and decreased to a low level after 6 hrs (Fig. 4B). It was noted that total AKT protein was up‐regulated at 3 hrs and maintained at high levels by 24 hrs. Correspondingly, phospho‐mTOR and phospho‐4EBP1 were pronouncedly increased by TSA at 6 hrs and sharply fell to very low levels after 9 hrs, in agreement with the results that phospho‐eIF2a accumulated after 9‐hrs treatments, which inactivated cap‐dependent translation (Fig. 3A). The data indicated that the TSA initially increased protein translation in a short time and subsequently caused the ER overloading.

To validate the role of the AKT/mTOR in TSA‐mediated effects, we blocked the AKT/mTOR pathway with PI3K inhibitor LY294002 (20 μM). Phospho‐AKT was significantly abrogated by LY294002 in combination with TSA (Fig. 4C), and cell viability in combined treatment rose to 74.04% compared to TSA treatment alone (29.18%) (Fig. 4D). Simultaneously, the elevated PARP cleavage by TSA was significantly alleviated in the co‐treatment (Fig. 4D). Similarly, overexpression of AKT1‐DN, which can impair AKT activity, also pronouncedly reversed the apoptosis induced by TSA, as demonstrated by the reduction in the PARP cleavage and restoration of cell survival (Fig. 4E, F).

Given the observations that activation of AKT/mTOR signalling and induction of ER stress were critical for TSA‐mediated cell death, a fine‐tuning investigation was required for addressing the association between these pathways and ER stress gene expression. As shown in Figure 4G, activation of AKT was clearly visible in TSA‐treated cells after 30 min. and peaked at 3 hrs. Accordingly, phospho‐4EBP1 was elevated from 30 min. to 3 hrs, subsequently leading to the induction of GRP78 and ATF3 at 1 hr and, thereafter in treated cells, consistent with the results in Figures 3A and 4B. These results implicated that rapid activation of PI3K‐AKT‐mTOR by TSA facilitated protein synthesis, subsequently triggered ER stress and apoptosis. The results in Figure 4H further supported the notion that inactivation of PI3K‐AKT by LY294002 led to the attenuated ER stress as demonstrated by decreased GRP78 and ATF3 in TSA‐treated cells. Thus, the transient activation of AKT/mTOR signalling that resulted in enhancement of protein synthesis contributed to the induction of ER stress and apoptosis in TSA‐mediated cytotoxicity.

### High HDAC activity and hyper‐acetylation contribute to chemo‐resistance in PCa

**Figure 5:**
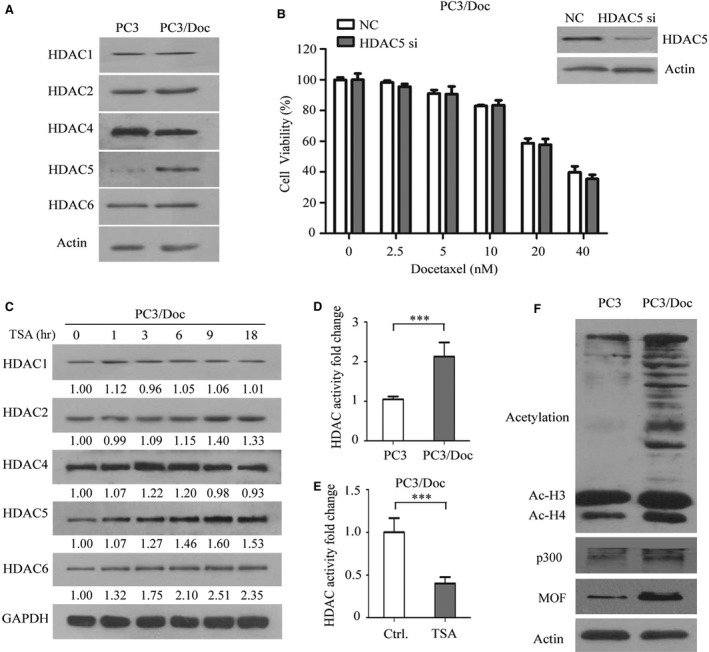
Hyper‐acetylation, but not HDAC5, contributes to chemo‐resistance in PCa. (**A**) Western blot analyses of HDAC family protein expression differences in two cell lines. (**B**) Knock‐down HDAC5 by siRNA in PC3/Doc cells, cell proliferation assay was monitored by MTT assay in response to the different concentration of docetaxel for 48 hrs. (**C**) Analysis of the effect of 0.4 μM TSA on the HDAC family by Western blot analysis. (**D**) The total HDAC activity was measured, in resistant cells compared to parent cells. ***P <* *0.01 compared with PC3 cells. (**E**) The total HDAC activity was measured with or without 0.4 μM TSA treatment for 9 hrs in PC3/Doc cells. ***P <* *0.001 compared with the negative control. (**F**) Western blot analyses of the acetylation status of the total protein, p300 and MOF in PC3 and PC3/Doc cells.

TSA acts as an HDAC inhibitor that promotes histone protein acetylation, which is an essential process in regulating chromatin topology and gene transcription. We next investigated whether the differential sensitivity of PC3 and resistant PC3/Doc cells to TSA was attributable to HDAC activity and the histone acetylation status. The results in Figure 5A showed that there were no detectable changes in the protein levels of HDAC1, HDAC2, HDAC4 and HDAC6, but the HDAC5 level was higher in PC3/Doc cells than PC3 cells. It was reported that HDAC5 depletion increases the efficacy of chemotherapeutics 22. Knock‐down of HDAC5 by siRNA did not increase the sensitivity of resistant cells to docetaxel (Fig. 5B), suggesting that HDAC5 might not be critical in chemo‐resistance in PC3/Doc cells. To determine if TSA affected the HDAC expression, time kinetic changes of HDACs were investigated in resistant cells. TSA treatment, to some extent, caused increases in the expression of HDAC2, 5 & 6 (Fig. 5C). Further study demonstrated that the HDAC activity in PC3/Doc cells was 2.13‐fold higher than that in PC3 cells and the activity reduced to one‐third by TSA (Fig. 5D, E), indicating that TSA affected HDAC activity instead of the expressions in resistant cells. Analysis of acetylation status revealed that the acetylated H3, particularly acetylated H4, was higher in the resistant cells than that of PC3 cells (Fig. 5F). Interestingly, hyper‐acetylation of non‐histone proteins was clearly present in the resistant cells as well (Fig. 5F). The changes in acetylation were also examined in tumour tissues, developed from animals bearing PC3 cells that were received docetaxel for 2 weeks (referred to as Doc1), and the tumours from treated mice were subsequently reseeded in mice to receive docetaxel for second‐round therapy (referred to as Doc2). The acetylation level of non‐histone proteins and histones was increased in Doc1 and Doc2 (Fig. S3A), which is consistent with observations in cultured resistant PC3/Doc cells (Fig. 5F). We next investigated whether histone acetyltransferases, for example, p300 or males absent on the first (MOF), are responsible for the enhancement of acetylated histone in resistant cells when HDAC activity was high. As shown in Figure 5F, the level of p300 and MOF was higher in resistant PC3/Doc cells than that of PC3 cells. Thus, induction of drug resistance was associated with enhanced protein acetylation in PCa cells.

We further investigated whether TSA effectively overcomes MDR in other cancer cells. Changes in acetylation and HDAC activity were examined in other multidrug‐resistant cell lines and their paired cell lines. The results in Figure S3B, C showed that there were increasing acetylation status and HDAC activity in vincristine‐induced oral cancer resistant tumour KB/VCR and docetaxel‐induced prostate cancer DU145/Doc cells, but not in resistant H460/RT cells and EC109/CDDP compared to the paired cells. Drug sensitivity analysis revealed that the resistant DU145/Doc cells and KB/VCR cells displayed, to some extent, more sensitive to TSA than their chemo‐sensitive parental cells, while this differential effect of TSA did not show on EC109/CDDP and EC109 cells, and H460/RT and H460 cells (Table S4), indicating that TSA had no advantage of reversal resistance in the chemo‐resistant cells with low acetylation and low HDAC activity.

### TSA increases histone acetylation and exacerbates chemo‐resistant cell death

**Figure 6:**
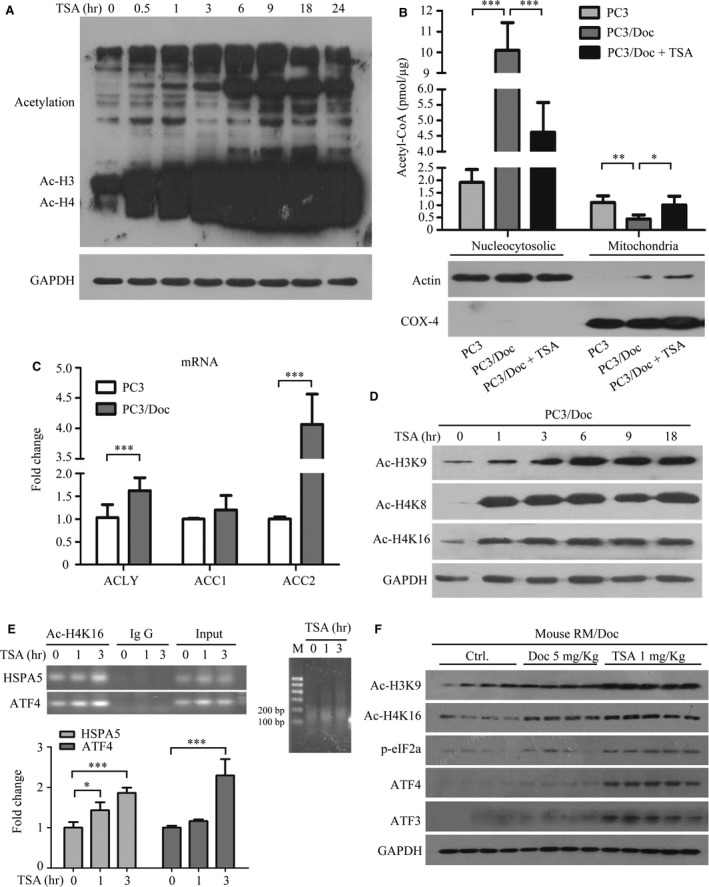
TSA increases histone acetylation and exacerbates chemo‐resistant death of cells that are hyper‐acetylated. (**A**) Analysis of the acetylation change of protein treated with 0.4 μM TSA in PC3/Doc cells. (**B**) Analysis of the acetyl‐CoA level of different organelles in PC3 and PC3/Doc cells with or without 0.4 μM TSA treatment for 9 hrs. (**C**) The acetyl‐CoA‐related candidate genes were evaluated by quantitative PCR assays. (**D**) TSA treatment results increase histone acetylation. (**E**) TSA‐induced increase in H4K16 acetylation at the HSPA5 and ATF4 proximal promoter. The primer sets, shown in the Materials and methods. H4K16 acetylation level at the GAPDH promoter was used as a control. The data are represented as the averages ± S.D. for at least three samples. *P <* *0.05 compared with the negative control. (**F**) Western blot analyses of the acetylation status of the total protein and proteins associated with ER stress in different treatment groups.

As acetylated H3 and H4 are key regulators that increase transcriptional activity, we reasoned that the effect of TSA on histone acetylation robustly alters gene transcription, which subsequently facilitates resistant cell lethality. As expected, elevations in the acetylated proteins and acetylated H3 and H4 were predominantly observed in response to TSA (upper panel in Fig. 6A). Because acetyl‐CoA is essential for histone and non‐histone protein acetylation 23, we, therefore, determined the levels of acetyl‐CoA in resistant cells. The results in Figure 6B showed acetyl‐CoA predominantly accumulated in the nucleocytosolic in resistant cells compared to the parent cells, whereas a low level of acetyl‐CoA was shown in mitochondria apparatus. On exposure to TSA, the acetyl‐CoA level in nucleocytosolic was sharply decreased in treated PC3/Doc cells (Fig. 6B), suggesting that acetyl‐CoA in the nucleocytosolic was shifted for protein acetylation in response to TSA. Furthermore, the results in Figure 6C clearly revealed that ATP‐citrate lyases (ACLY), a key enzyme for generating cytosolic acetyl‐CoA, were markedly increased in resistant cells. Similarly, enhanced acetyl‐CoA carboxylases (ACC1 and ACC2), which utilized acetyl‐CoA as a building block for fatty acid synthesis, were observed in PC3/Doc cells (Fig. 6C), supporting the importance of nucleocytosolic acetyl‐CoA in promoting protein acetylation and fatty acid synthesis.

We next examined changes of several critical Lys in histones, which have been identified to regulate gene transcriptions, in response to TSA. As shown in Figure 6D, changes in Ac‐H3K9, Ac‐H4K8 and Ac‐H4K16 were evident in response to TSA. ChIP experiments, with an antibody detecting Ac‐H4K16, followed by PCR revealed an increase of Ac‐H4K16 on the promoter of HSP5 (gene coded for GRP78 protein) and ATF4 in TSA‐treated cells (Fig. 6E). The insert RT‐PCR showed the preparation efficiency of mono‐nucleosome DNA templates digested by the nuclease. Re‐analysis of these key molecules in tumour tissues indicated that phosphor‐eIF2α, ATF3 and ATF4 were remarkably induced in TSA‐treated mice (Fig. 6F), in agreement with the results observed in cultured cells (Fig. 3A). Both Ac‐H4K16 and Ac‐H3K9 were also increased in TSA‐treated mice, while they changed slightly in response to vehicle or docetaxel (Fig. 6F). These results clearly demonstrated that TSA‐initiated acetylation, activating transcription of ER stress‐responsive genes, was critical for exacerbating chemo‐resistant cell death.

## Discussion

In this study, we reported a novel finding that docetaxel‐induced CRPC chemo‐resistant cells were hyper‐acetylated, and an HDAC inhibitor TSA induced resistant cell death *via* caspase‐dependent apoptosis with IC50 at 0.33 ± 0.06 μM compared with the paired PCa cells (2.87 ± 0.21 μM). Enhancement of gene expressions and induction of the ER stress by TSA played a critical role in facilitating resistant cell death. TSA markedly increased histone acetylation, resulting in transcriptional activation of gene expressions, including ER stress‐responsive genes. Additionally, TSA transiently activated AKT/mTOR signalling, leading to the enhancement of protein translation. Therefore, transcription or translation inhibition and knock‐down of ATF3 almost completely reversed TSA‐induced apoptosis. The molecular mechanisms underlying TSA efficiency displayed that higher nucleocytoplasmic acetyl‐CoA was responsible for elevated acetylation status of proteins, and the presence of HAT and HDAC activity maintains acetylation balance in chemotherapy‐resistant PCa cells.

It has been demonstrated that the HDACs are abundantly expressed and up‐regulated in PCa 5, 24 and play a critical role in regulating the androgen receptor 25. Therefore, HDACs offer a therapeutic target to inhibit PCa proliferation, and HDACi are now being evaluated for their effects on CRPC patients 26. For example, SAHA attenuated chemo‐resistance in biliary tract cancer 27, although the mechanism requires further investigation. In addition to the regulatory effect on histone acetylation, HADCi has the ability to effectively promote cell apoptosis by reducing P‐glycoprotein (P‐gp) expression that is highly expressed in MDR cells 8, 28. Our study and others have demonstrated that docetaxel‐induced multidrug‐resistant PCa cells have no P‐gp expression, and multiple mechanisms contribute to the acquired MDR 29, 30, 31. This study revealed that multidrug resistance protein 2 (MRP2), not P‐gp, was overexpressed in PC3/Doc cells. A detailed analysis of MRP2 in conferring drug resistance and response to TSA in multidrug‐resistant PCa cells requires further investigation.

The effect of TSA on histone acetylation was demonstrated; for example, H4K16 acetylation contributes to the rapid up‐regulation of GRP78 and ATF4 and activation of AKT/4EBP1, which in turn results in severe ER stress and cell death. Interestingly, our results demonstrated that drug‐resistant cells were hyper‐acetylated compared to the drug‐sensitive counterparts, although high HDAC activity was observed in resistant cells. Enhanced p300 and MOF, at least in part, may contribute to the histone acetylation in resistant cells, and further investigation is required. Studies have provided evidence that histone acetylation results in drug resistance to methotrexate in choroid plexus carcinomas 32. Wang *et al*. report that enhanced HDAC activity is shown in cisplatin‐enriching cancer stem cells and paclitaxel‐resistant cells, which confer multidrug resistance in non‐small cell lung cancer 33, 34. However, they found a decrease in acetylated H3 and acetylated H4 levels associated with high HDAC activity. In addition, hyper‐acetylation of histones enhances anticancer activity of chemotherapeutic agents; for example, gefitinib‐resistant lung carcinoma cells are sensitive to GNPs (gefitinib‐loaded PLGA nanoparticles). GNPs are able to hyper‐acetylate histone H3 that may account for the augmented cell death, because inhibition of HATs decreases the anti‐proliferative activity of GNPs 35. Therefore, boosting acetylation with TSA speeded up transcription, resulting in more severe cellular stresses and facilitating cell death.

There was an accumulation of acetyl‐CoA in the nucleocytosolic and enhancement of ACLY, which is important for the proliferation of glycolytically converted tumour cells 36, dominantly present in resistant cells, supporting that active glucose metabolism and enhanced lipid metabolism occurred in resistant cells. Elevated acetylated non‐histone proteins were clearly observed in the cytosol, which might be ascribed to the high acetyl‐CoA, consistent with reports that high nucleocytoplasmic acetyl‐CoA levels favour its utilization in lipid synthesis, histone acetylation and non‐histone protein acetylation 37, 38. Our results also support that in response to TSA, the acetyl‐CoA level in mitochondria was increased, whereas the nucleocytoplasmic acetyl‐CoA sharply decreased and was accompanied with cell death. Further studies will be needed to elucidate the mechanism by which TSA modulates acetyl‐CoA production.

In summary, the levels of acetylation proteins and/or nuclear‐cytosolic acetyl‐CoA may act as biomarkers of HDACi response prediction in chemo‐resistant cancers. HDACi, such as TSA and SAHA, evolved as a potential strategy for overcoming drug resistance in PCa.

## Conflict of interest

The authors declare that there are no conflicts of interest.

## Supporting information


**Figure S1** (A‐F) Identification of different Chemotherapy drugs in docetaxel sensitive and resistant cells. The cell viability assays treated with or without Doxorubicin, Vincristine, Cisplatin, Docetaxel, Trichostatin A and SAHA in docetaxel‐sensitive and ‐resistant cells. (G) The expression of pro‐apoptotic BAX and anti‐apoptotic Bcl2 in TSA‐treated PC3/Doc cells was estimated by western blotting analysis. (H) Western blot analysed the expression of PARP in 1 μM TSA‐treated PC3 cells. (I‐L) Docetaxel inhibits tumor growth in the RM‐1 homotransplantations mouse model. (I) Body weight of mice was measured every 2 days after the indicated treatment (*n* = 4). (J) Representative tumors from the three groups are shown (Ctrl group: *n* = 3 and Doc group: *n* = 4). (K) Tumor volume from homografts in different treatment groups was recorded every 2 days. Data are represented as the mean ± S.E.M. (*n* = 4). *P < 0.05 compared with the negative control. (L) Tumor weight was detected at time of sacrifice for different treated‐groups. Data are shown as the mean ± S.E.M. (*n* = 4).

**Figure S2** TSA didn't induce death receptor pathway apoptosis or mitochondrial pathway apoptosis.

**Figure S3** The global protein acetylation level in tissue samples.

**Appendix S1** Materials and Methods.
**Table S1** RT‐PCR Primer.
**Table S2** Primer for ChIP.
**Table S3** Evaluation of cells resistant capability from the two generation tumors resistant to chemotherapy drugs.
**Table S4** TSA sensitivity was assessed on other drug sensitive and resistant cells.
