# Supplementary figures and images for: Hyper‐acetylation contributes to the sensitivity of chemo‐resistant prostate cancer cells to histone deacetylase inhibitor Trichostatin A
Source: J Cell Mol Med. 2018 Jan 12;22(3):1909–22. doi: 10.1111/jcmm.13475 (PMC5824406; doi:10.1111/jcmm.13475)

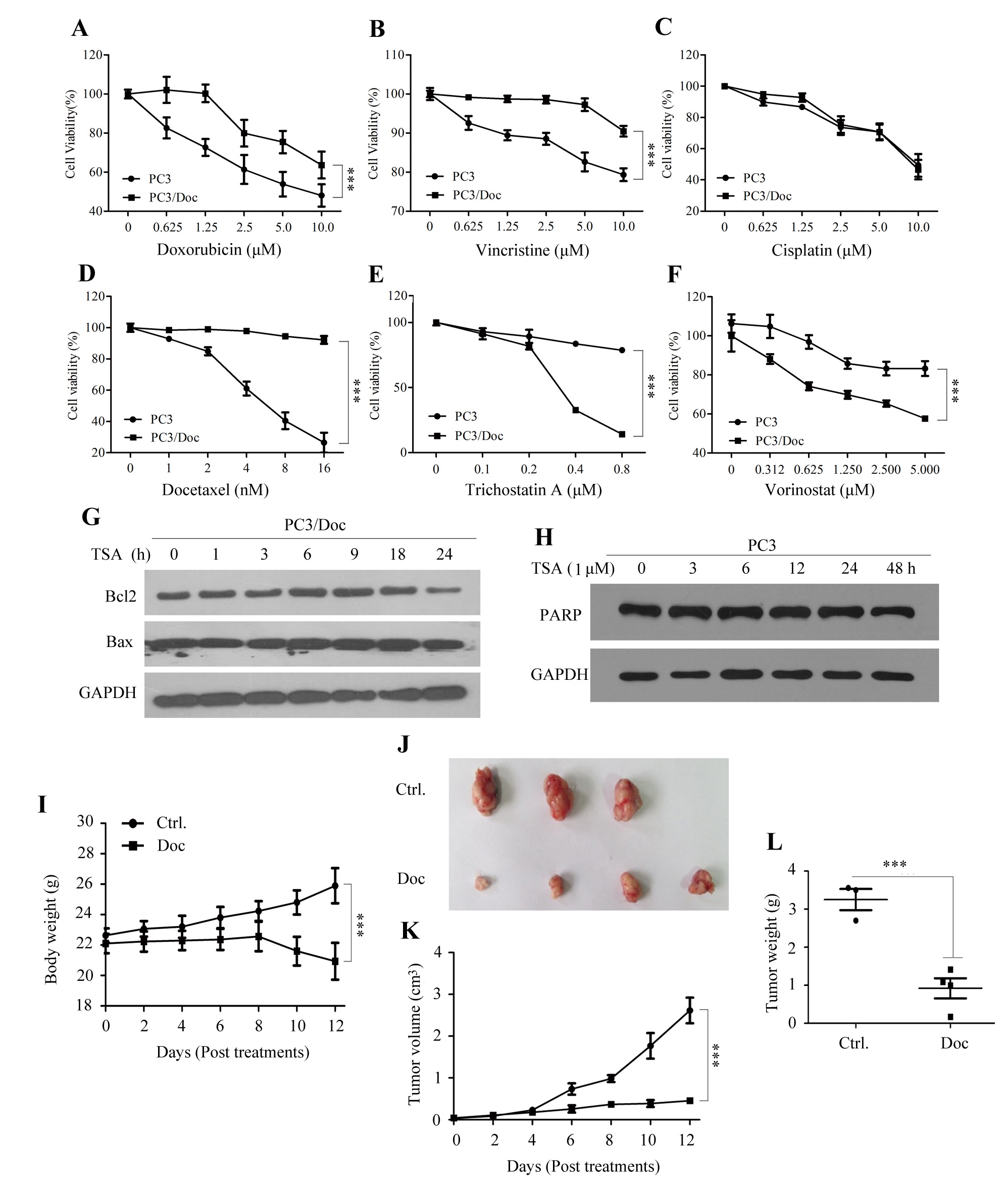

Supplement: Supplementary file 1 — Figure S1 (A‐F) Identification of different Chemotherapy drugs in docetaxel sensitive and resistant cells. The cell viability assays treated with or without Doxorubicin, Vincristine, Cisplatin, Docetaxel, Trichostatin A and SAHA in docetaxel‐sensitive and ‐resistant cells. (G) The expression of pro‐apoptotic BAX and anti‐apoptotic Bcl2 in TSA‐treated PC3/Doc cells was estimated by western blotting analysis. (H) Western blot analysed the expression of PARP in 1 μM TSA‐treated PC3 cells. (I‐L) Docetaxel inhibits tumor growth in the RM‐1 homotransplantations mouse model. (I) Body weight of mice was measured every 2 days after the indicated treatment (n = 4). (J) Representative tumors from the three groups are shown (Ctrl group: n = 3 and Doc group: n = 4). (K) Tumor volume from homografts in different treatment groups was recorded every 2 days. Data are represented as the mean ± S.E.M. (n = 4). *P < 0.05 compared with the negative control. (L) Tumor weight was detected at time of sacrifice for different treated‐groups. Data are shown as the mean ± S.E.M. (n = 4). [file JCMM-22-1909-s001.tif]

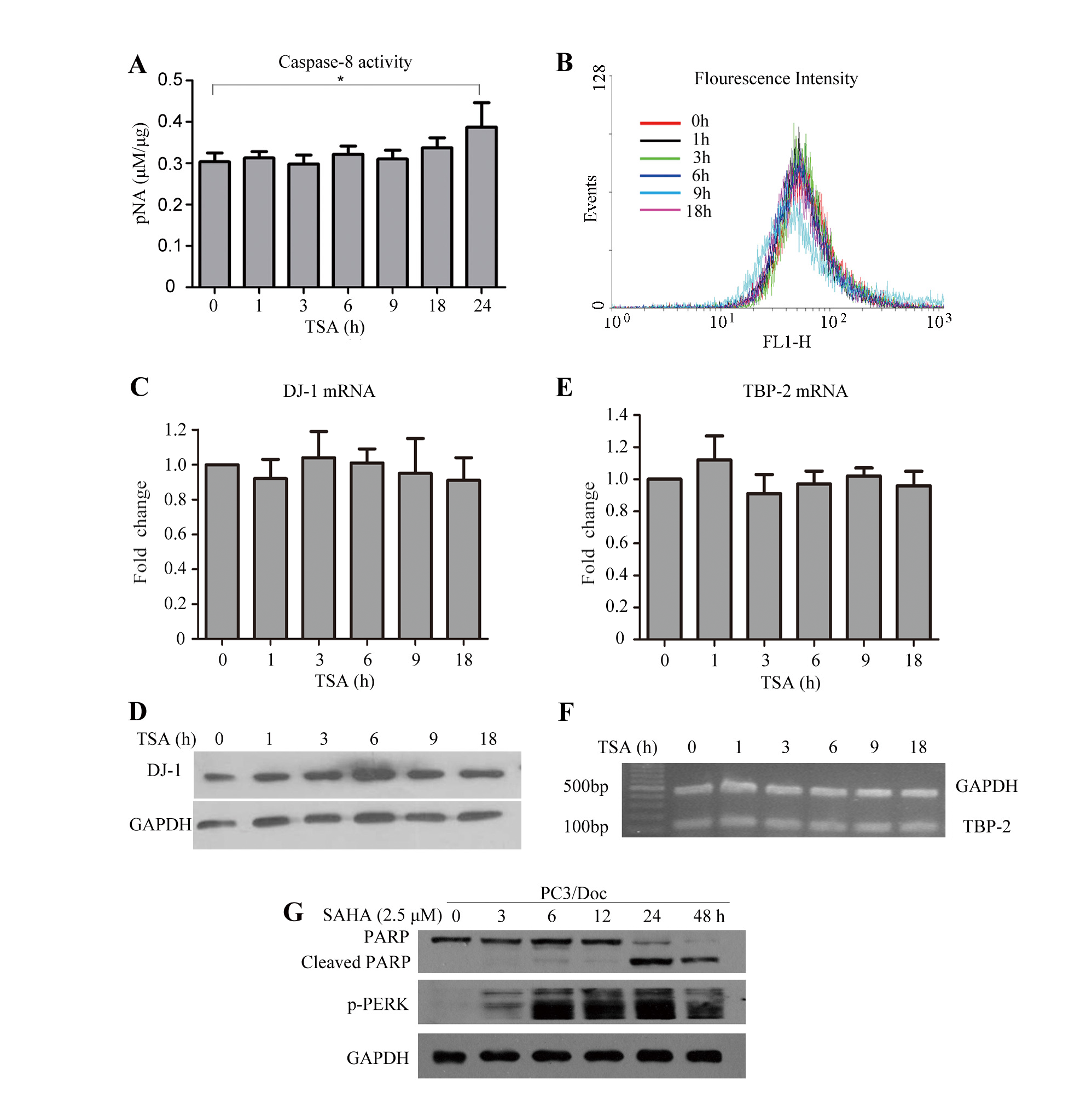

Supplement: Supplementary file 2 — Figure S2 TSA didn't induce death receptor pathway apoptosis or mitochondrial pathway apoptosis. [file JCMM-22-1909-s002.tif]

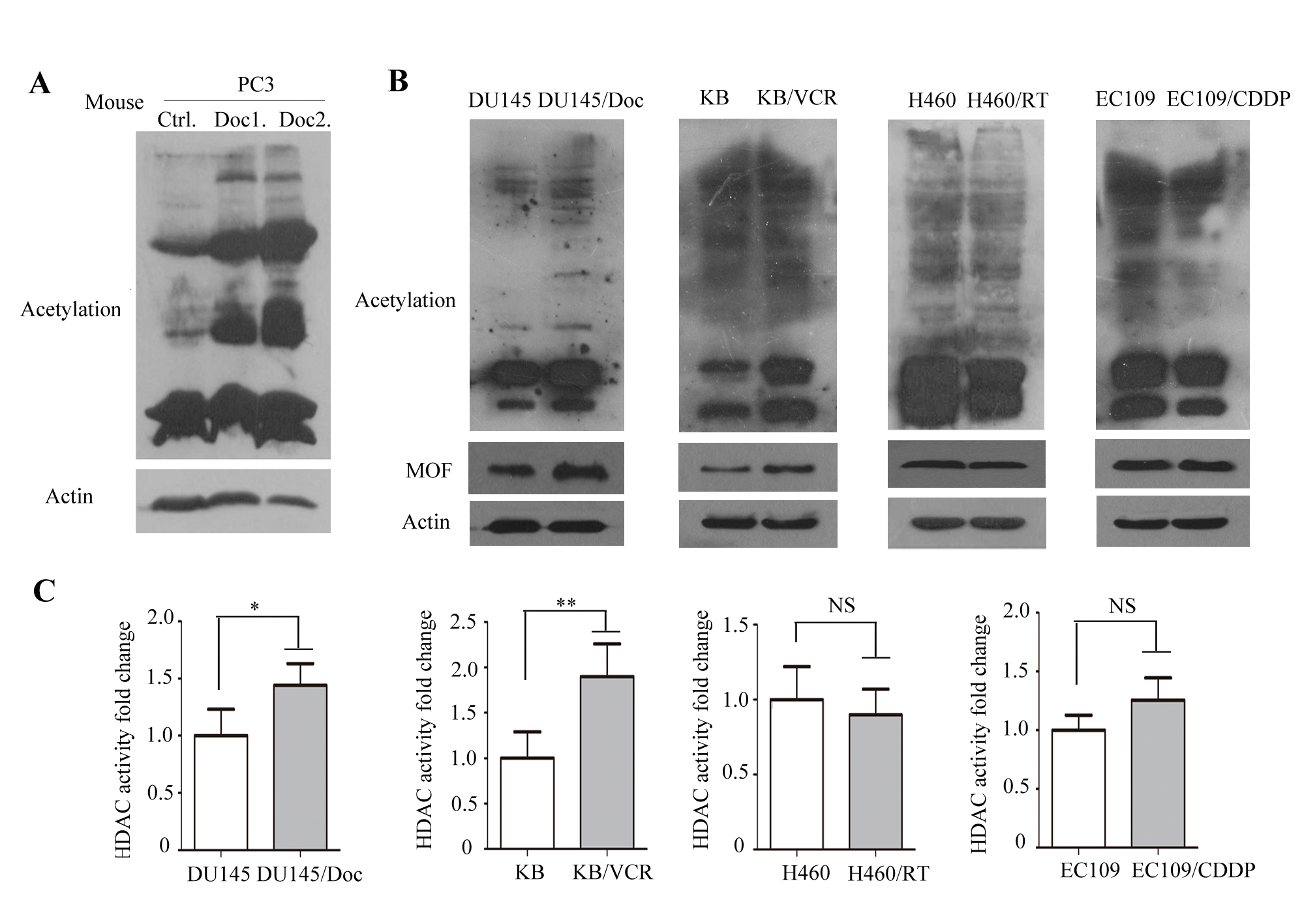

Supplement: Supplementary file 3 — Figure S3 The global protein acetylation level in tissue samples. [file JCMM-22-1909-s003.tif]
