# Supplementary material for: Hyper‐acetylation contributes to the sensitivity of chemo‐resistant prostate cancer cells to histone deacetylase inhibitor Trichostatin A
Source: J Cell Mol Med. 2018 Jan 12;22(3):1909–22. doi: 10.1111/jcmm.13475 (PMC5824406; doi:10.1111/jcmm.13475)
Supplement: Supplementary file 4 — Appendix S1 Materials and Methods. Table S1 RT‐PCR Primer. Table S2 Primer for ChIP. Table S3 Evaluation of cells resistant capability from the two generation tumors resistant to chemotherapy drugs. Table S4 TSA sensitivity was assessed on other drug sensitive and resistant cells. [file JCMM-22-1909-s004.docx]

**Supplementary Materials and Methods**

**Cell culture**

DU145/Doc cells were obtained by stepwise selection with increasing Docetaxel concentrations and maintained in culture from prostate cancer DU145 cells. Esophageal cancer EC109 and the cisplatin-resistant EC109/CDDP cells were a gift from the Second Hospital of Shandong University. All cells were cultured in RPMI 1640 medium (Hyclone) supplemented with 10% fetal bovine serum (GIBICO), 100 U/ml penicillin and 100 g/ml streptomycin.

**Chemicals**

Docetaxel (Aventis Pharma S.A., France), Cisplatin (Qilu Pharmaceutical co., LTD, China), Doxorubicin and Vincristine (Main Luck Pharmaceuticals Inc, Shenzhen, China) were purchased from the Second Hospital of Shangdong University.

**Determination of Caspase 8 activity**

Caspase-8 activity analyses were performed using a Caspase 8 Activity Assay Kit (Beyotime Biotechnology, China). After treatment with 0.4 mM TSA at desired times, all procedures were performed according to the manufacturer’s protocol.

**Determination of acetyl-Coenzyme A (Ac-CoA)**

Ac-CoA analyses were performed using a PicoProbe™Acetyl CoA Fluorometric assay kit (Biovision Life Science, USA). After treatment with 0.4 mM TSA at desired times, cell lysates were ground 30 times and centrifuged at 800 g for 10 min to discard precipitation. The supernatants were centrifuged at 1,2000 g for 20 min to separate the nucleocytoplasm and mitochondria. The remaining procedures were performed according to the manufacturer’s protocol. Fluorescence was measured using Ex/Em = 535/589 nm with a plate reader (Bio-Rad, USA).

**Determination of HDAC activity**

HDAC activity analyses were performed using a HDAC activity Quantitative Colorimetric Detection Kit (GENMED Scientifics Inc. U.S.A). All procedures were performed according to the manufacturer’s protocol.

**Determination of ROS**

Cellular ROS concentrations were measured with a flow cytometer (BD Biosciences) after incubating cells with a fluorescence probe 2, 7-dichlorofluorescein-diacetate (DCFH-DA, Sigma-Aldrich).

**To establish the multidrug resistance murine model loading PC3 cells**

Male athymic (BALB/c-nu) mice (4–6 weeks) were obtained from the Animal Center of China Academy of Medical Sciences (Beijing, China). The cells (1 x 107) were injected subcutaneously into the right flanks of the mice and allowed to establish tumors. When the tumor volume reached approximately 600 mm3, animals were given 5 mg/kg docetaxel intraperitoneal injections every 2 days for 2 weeks (referred to as Doc1), and the tumors from docetaxel mice were subsequently reseeded in mice for docetaxel second-round therapy (referred to as Doc2). Mice were given PBS as a vehicle control. The resistant capability from the two generated tumors to chemotherapy drugs was evaluated in Supplementary Table 3.

**Supplementary figures**

**Sup. 1** (A-F) Identification of different Chemotherapy drugs in docetaxel sensitive and resistant cells. The cell viability assays treated with or without Doxorubicin, Vincristine, Cisplatin, Docetaxel, Trichostatin A and SAHA in docetaxel-sensitive and -resistant cells. (G) The expression of pro-apoptotic BAX and anti-apoptotic Bcl2 in TSA-treated PC3/Doc cells was estimated by western blotting analysis. (H) Western blot analysed the expression of PARP in 1 μM TSA-treated PC3 cells. (I-L) Docetaxel inhibits tumor growth in the RM-1 homotransplantations mouse model. (I) Body weight of mice was measured every 2 days after the indicated treatment (n = 4). (J) Representative tumors from the three groups are shown (Ctrl group: n = 3 and Doc group: n = 4). (K) Tumor volume from homografts in different treatment groups was recorded every 2 days. Data are represented as the mean ± S.E.M. (n = 4). *P < 0.05 compared with the negative control. (L) Tumor weight was detected at time of sacrifice for different treated-groups. Data are shown as the mean ± S.E.M. (n = 4).

**Sup. 2** TSA didn't induce death receptor pathway apoptosis or mitochondrial pathway apoptosis. (A) Caspase-8 activity was analysed in 0.4 μM TSA-treated PC3/Doc cells in a time-dependent phase. (B) Effect of 0.4 μM TSA on intracellular ROS formation in PC3/Doc cells assayed by ﬂow cytometry stained with H(2)DCFDA at different time points. (C) (D) QRT-PCR and western blotting analysis of mRNA (C) and protein (D) levels of oxidative stress response protein DJ-1 in TSA-treated PC3/Doc cells. GAPDH served as a loading control. (E) (F) QPCR and RT-PCR analyses the mRNA expression of TBP-2 in TSA-treated PC3/Doc cells. (G) Western blot analysis of PARP and p-PERK levels in whole cell lysates from PC3/Doc cells treated with SAHA for the indicated times.

**Sup. 3** The global protein acetylation level in tissue samples. (A) Analysis of the acetylation level in 3 generations of tumors resistant to docetaxel by western blot analysis. (B) Western blot analyses of the acetylation status on other chemo-resistant cells and the parental cells. (C) HDAC activity were evaluated in drug sensitive and resistant cell lines.

**Supplementary Tables**

**Table 1**. RT-PCR Primer

| **Gene** | **Forward** | **Reverse** |
| --- | --- | --- |
| CHOP | ATCTGCTTTCAGGTGTGGTG | AGAGCCAAAATCAGAGCTGG |
| ACLY | GCAGCATCGCAAACTTCAC | CTTCCCGACTTCTCCCATC |
| ACC1 | TGGATGATGGGCTACAGGC | CACCTCCAGATGACGAGATTG |
| ACC2 | GAGATGTTCCGCAACGAGC | TTGGCAATGTCCACAATCAG |
| 18s | GGACACGGACAGGATTGAC | CGGACATCTAAGGGCATCA |
| DJ-1 | CCTACTGCTCTGTTGGCTCAT | CGCAAACTCGAAGCTGGTC |
| TPB-2 | CCAGACCAAGGTGCTGACT | GATGTTGCAGCCCAGGATAG |
| GAPDH | GCCTCAAGATCATCAGCAATG | GTCAAAGGTGGAGGAGTGG |

**Table 2**. Primer for ChIP.

| **Gene** | **Forward** | **Reverse** |
| --- | --- | --- |
| HSAP5 | TTCGCGAGTGTGAGAGGGAAG | GTAGCACAGGAGCACAGCGCA |
| ATF4 | ACTGCGCTGACACCGGAAGCGA | CCTCACGAAAGGAGAGAGGTGT |

**Table 3**. Evaluation of cells resistant capability from the two generation tumors resistant to chemotherapy drugs.

| IC50 (μM) | Doc. 0 | Doc.1 | Doc.2 |
| --- | --- | --- | --- |
| Dorubicin | 0.99 ± 0.15 | 0.87 ± 0.19 | 1.78 ± 0.26 |
| Vincristine | 0.47 ± 0.08 | 0.94 ±0.23 | 0.64 ± 0.31 |
| Cis-platinum | 10.46 ± 0.97 | 11.50 ± 0.58 | 11.17 ± 0.75 |
| Docetaxel (nm) | 12.73 ± 2.89 | 79.90 ± 4.91 | 77.94 ± 3.52 |
| Trichostatin A | 2.49 ± 0.53 | 1.20 ±0.37 | 1.16 ± 0.26 |

**Table 4.** TSA sensitivity was assessed on other drug sensitive and resistant cells.

| IC50 | TSA ((μM) |
| --- | --- |
| DU145 | 2.56 ± 0.32 |
| DU145/Doc | 1.89 ± 0.19 |
| KB | 1.48 ± 0.27 |
| KB/VCR | 0.62 ± 0.14 |
| H460 | 2.00 ± 0.36 |
| H460/RT | 1.94 ± 0.45 |
| EC109 | 1.48 ± 0.31 |
| EC109/CDDP | 1.20 ± 0.21 |
